# Supplementary material for: Re-emergence and influencing factors of mountain-type zoonotic visceral leishmaniasis in the extension region of Loess Plateau, China
Source: PLoS Negl Trop Dis. 2024 May 31;18(5):e0012182. doi: 10.1371/journal.pntd.0012182 (PMC11168655; doi:10.1371/journal.pntd.0012182)
Supplement: Attachment — Submitted filename: A letter with responses.docx [file pntd.0012182.s004.docx]

Dear editors and reviewers:

Thank you for your letter and for the reviewers’ comments concerning our manuscript entitled “Re-emergence and influencing factors of mountain-type zoonotic visceral leishmaniasis in the extension region of Loess Plateau, China” (ID: PNTD-D-23-01322). Those comments are all valuable and very helpful for revising and improving our paper, as well as the important guiding significance to our researches. We have studied comments carefully and have made correction which we hope meet with approval.

Revised portion are marked in red in the paper. The main corrections in the paper and the responds to the reviewer’s comments are as flowing:

**Responds to the reviewer’s comments:**

Reviewer #1:

**Methods**

**Q1**: The objectives of the study clearly and study design appropriate, the sample size sufficient.

R: Thank you for acknowledging the clarity of our study objectives and the appropriateness of our design, as well as recognizing the sufficiency of our sample size. Your positive feedback validates our methodology and approach.

**Results**

(No Response)

**Conclusions**

(No Response)

**Editorial and Data Presentation Modifications?**

(No Response)

**Summary and General Comments**

Q2: In this manuscript, the variables have been chosen correctly and their analysis has been done accurately. But it has no novelty, and in my opinion, it does not have the necessary quality to be published in Plus One journal.

R: Thank you for your valuable feedback. While we acknowledge the concern regarding novelty, it's crucial to highlight the necessity of our study, particularly given the rising trend of mountain-type zoonotic visceral leishmaniasis in China. Investigating the factors contributing to the resurgence of this epidemic is paramount. Despite employing conventional case-control methods, our other studies utilizes classic methodologies and integrates sophisticated analyses such as spatiotemporal analysis and ecological niche modeling to explore the influences of meteorological, geographical, and economic factors [1,2]. While those approaches offer a macroscopic view, the case-control methodology provides a microscopic perspective from the standpoint of both patients and healthy individuals. This study serves as a preliminary investigation, and future research endeavors will aim to refine indicators and elucidate specific contributing factors further.

**Reviewer #2:**

**Methods**

Yes

**Results**

Yes

**Conclusions**

Yes

**Editorial and Data Presentation Modifications?**

Q1: Dear Author, would you please confirm that you interviewed the patients and control at the same time?

R: We confirm that for our study, the patient group was interviewed separately from the control group. Control subjects were selected strictly based on proximity to the patient's residential area, ensuring similarity in gender and age within a three-year range. Details regarding the selection process can be found in the 'kala-azar' column of the compiled table, and all survey data was entered into Epidata for analysis.

**Summary and General Comments**

Q2: In general it is fine

Reviewer #3:

**Methods**

Q: Thank you for providing detail description of the methodology. I have a few questions as following:

- Page 10, Line 3 "Population data were collected from the Shanxi Statistical...." it is control group, right? if so, you have to give more details about sampling inclusion criteria/ sampling technique.

R: Thank you for your review comments. The population data mentioned here is used to calculate the incidence rate of MT-ZVL in Yangquan City from 2006 to 2021 (number of cases/population). Detailed information regarding the sampling inclusion and exclusion criteria can be found in the ‘Identification of influencing factors for the rapid rise in MT-ZVL epidemics’ section, specifically in lines 161-163. We conducted a case-control study where MT-ZVL cases were matched at a ratio of 1:3 with non-MT-ZVL patients…”

- Page 10, It is mentioned that the diagnosis done according to the "National Diagnostic Criteria for Visceral Leishmaniasis in China." would you please add a reference for that.

R: Thank you for your suggestion. We have added a reference for the ‘National Diagnostic Criteria for Visceral Leishmaniasis in China’ in the relevant section.

**Results**

Q: In page 13, the map, what is the indications for the other colors not mentioned in the map legend. (Yellow and blue)

R: The yellow and blue colors represent different subdivisions within Yangquan City, at a smaller geographical scale. We have made modifications and additions to the map legend to clarify this.

- Table 1, did you go with the questionnaire with population less than 5 years old, 15 years old? please confirm.

R: The questionnaire survey included cases of children aged below five and fifteen years old. Specific numbers are clearly outlined in Table 1. This information has been verified.

- Table 2, the majority of cases did not hear about the disease before, it means that they are not aware of their diagnosis or there is different between the name in the survey and the one use in the community? please explain.

R: Yes, we have considered this issue. The specific name of the disease is quite technical, and during the survey, we collaborated with local CDC personnel to ensure that information was conveyed in understandable language. However, many cases were scattered in rural areas, mainly among the elderly population, where health education and awareness about the disease are relatively low. Hence, they indeed lack awareness about this particular illness.

- Table 2, why did you include the cases and control who responded about "have you heard about the MT-ZVL" if they say "No" in the analysis of the rest of questions. then the answer after that might be randomly and not reflecting the reality, right? please explain

R: Thank you for your question. We included cases and controls who responded ‘No’ to the question about whether they had heard about MT-ZVL in order to assess their baseline knowledge and awareness of the disease. While it’s true that their responses to subsequent questions may not fully reflect their understanding of the disease, including this question allows us to examine any differences in knowledge and perceptions between those who have heard about MT-ZVL and those who have not.

**Conclusions**

In the limitation section. we may discuss that the time frame of cases was very wide and that might affect the knowledge as well as the living condition including the risk factors.

- Also, the mobility of cases over 15 years can do the same.

R: Thank you for your suggestion. We have incorporated the mentioned points into the limitations section. We will address the broad timeframe of cases, acknowledging its potential impact on knowledge and living conditions, including risk factors.

- Page 20, The author mentioned "The awareness of MT-ZVL knowledge was 59.8% ..." Would you explain how it is calculated as it was not mentioned before and not presented in the results.

R: I sincerely apologize for the oversight. It appears that due to rounding, the calculation in Table 1 mistakenly rounded 59.8 to 60.0. We deeply regret this error and have taken immediate steps to rectify it.

**Editorial and Data Presentation Modifications?**

(No Response)

**Summary and General Comments**

Thank you very much for the effort done in the paper and the level of scientific investigations for answering the objective of the study. Generally, the paper is well organized and homogenous even there was some comments to be answered mentioned above in different sections as well as some common points mentioned below

- In the Abstract methods. ot is confusing for me particularly with control part

R: We review the abstract methods section to ensure clarity, particularly regarding the control group.

- In the Abstract results. it would be better to quantify the results mentioned (Adding numbers) to the narrative paragraph.

R: We appreciate your suggestion. We will enhance the abstract by quantifying the results mentioned, adding specific numbers to the narrative paragraph.

- In introduction, in the second paragraph, please add (In China) as you were talking globally just in the previous sentences.

R: Thank you for pointing that out. We will make the necessary clarification by adding "(In China)" in the second paragraph to indicate the geographical scope.

- Regarding the declaration of ethics approval and consent to participate. this research has 2 parts of data collection. The first part is 2ry analysis and the second part is active data collection. Please double check if there is no need for IRB approval.

R: We will thoroughly review the research protocol to ensure compliance with ethical standards, especially considering the two parts of data collection. If needed, we will seek IRB approval for the active data collection part.

**REFERENCES:**

1.Li Y, Luo Z, Hao Y, et al. Epidemiological features and spatial-temporal clustering of visceral leishmaniasis in mainland China from 2019 to 2021. Front Microbiol. 2022;13:959901.

2.Hao Y, Luo Z, Zhao J, Gong Y, Li Y, Zhu Z, Tian T, Wang Q, Zhang Y, Zhou Z, et al. Transmission Risk Prediction and Evaluation of Mountain-Type Zoonotic Visceral Leishmaniasis in China Based on Climatic and Environmental Variables. Atmosphere. 2022; 13(6):964.
